# Mackerel-Derived Fermented Fish Oil Promotes Hair Growth by Anagen-Stimulating Pathways

**DOI:** 10.3390/ijms19092770

**Published:** 2018-09-14

**Authors:** Jung-Il Kang, Hoon-Seok Yoon, Sung Min Kim, Jeong Eon Park, Yu Jae Hyun, Ara Ko, Yong-Seok Ahn, Young Sang Koh, Jin Won Hyun, Eun-Sook Yoo, Hee-Kyoung Kang

**Affiliations:** 1 Department of Medicine, School of Medicine, Jeju National University, 102 Jejudaehakno, Jeju 63243, Korea; jikang0024@jejunu.ac.kr (J.-I.K.); hsyoon717@jejunu.ac.kr (H.-S.Y.); seongmin85@naver.com (S.M.K.); jeongeon7780@hanmail.net (J.E.P.); yujae1113@jejunu.ac.kr (Y.J.H.); rhdkfk0123@naver.com (A.K.); yskoh7@jejunu.ac.kr (Y.S.K.); jinwonh@jejunu.ac.kr (J.W.H.); eunsyoo@jejunu.ac.kr (E.-S.Y.); 2 Choung Ryong Fisheries Co. Ltd., 7825 Iljudong-ro, Namwon-epu, Seogwipo, Jeju 63612, Korea; ecoil@hanmail.net; 3 Jeju Research Center for Natural Medicine, Jeju National University, 102 Jejudaehakno, Jeju 63243, Korea

**Keywords:** mackerel-derived fermented fish oil, docosahexaenoic acid, hair growth, dermal papilla cells, anagen, cell cycle progression, extracellular signal–regulated kinase, p38, Akt, β-catenin

## Abstract

Hair growth is regulated by the interaction between dermal papilla cells (DPC) and other cells inside the hair follicle. Here, we show the effect and action mechanism of mackerel-derived fermented fish oil (FFO) extract and its component docosahexaenoic acid (DHA) in the control of hair growth. The hair growth effect of FFO extract was evaluated by the culture method of vibrissa follicles and in vivo dotmatrix planimetry method. FFO extract increased the length of hair-fibers and enabled stimulated initiation into the anagen phase of the hair cycle. As expected, FFO extract significantly increased DPC proliferation. FFO extract induced the progression of the cell cycle and the activation of extracellular signal-regulated kinase (ERK), p38 and Akt. FFO extract induced nuclear translocation of β-catenin, a stimulator of anagen phase, through an increase of phospho-glycogen synthase kinase3β (GSK3β) level. Since various prostaglandins are known to promote hair growth in humans and mice, we examined the effect of DHA, a main omega-3 fatty acid of FFO, on DPC proliferation. DHA not only increased DPC proliferation but also upregulated levels of cell cycle-associated proteins such as cyclin D1 and cdc2 p34. These results show that FFO extract and DHA promote hair growth through the anagen-activating pathways in DPC.

## 1. Introduction

Hair loss is caused by various factors, including stress hormones, chemotherapy and insufficient nutrition, and is a serious problem for modern people regarding appearance [1,2,3,4]. Hair transplantation and growth factor injection can be used to treat hair loss [5,6], but so far only two drugs (minoxidil and finasteride) have been approved by the Food and Drug Administration for treatment [7,8]. Minoxidil, a ATP-sensitive potassium (K_ATP_) channel opener, was originally developed and used as a therapeutic agent for hypertension [9], but has also been reported to be useful for the treatment of hair loss [7]. Finasteride is known to be used not only in the treatment of prostatic hyperplasia but also in the improvement of androgenetic hair loss, which is the most prevalent cause for hair loss [8,10]. However, the two drugs have problems such as a temporary effect or restrictions on use in women [11,12]. Thus, there is an increasing demand for drugs that have reduced side effects compared to those of existing drugs or for materials without side effects.

Human hair undergoes the hair cycle of growth phase (anagen), regression phase (catagen) and resting phase (telogen) for a lifetime. Therefore, controlling the hair cycle through maintaining anagen or shortening catagen and telogen and promoting the progression to anagen in hair growth is considered important [13]. Interactions between adjacent cells in the hair follicles (dermal papilla cells (DPC), dermal sheath cells, stem cells, and hair germ cells) during changes of the hair cycle regulate hair growth or hair loss [13]. The dermal papilla, a mesenchymal derived fibroblast, is thought to be the main regulator of the hair cycle, which, similar to hair follicles stem cells, is always present, unlike the matrix cells surrounding dermal papilla [14]. On the other hand, activation of wnt/β-catenin is thought to increase the proliferation of follicular cells such as DPC and induce hair growth in vivo [15,16]. In fact, minoxidil increased the duration of hair growth in vivo, which may be due to the activation of wnt/β-catenin in dermal papilla [15]. The loss of β-catenin, a component of wnt/β-catenin, resulted in severe hair loss [17], indicating that wnt/β-catenin signaling directly or indirectly affects hair growth as well as growth of hair follicles cells. It is known that cell proliferation is closely related to the progression of the cell cycle and the level of cell cycle-associated proteins, including cyclins, cyclin-dependent kinases (CDKs), retinoblastoma protein (pRB), and CDK inhibitors [18]. Minoxidil has been shown to induce proliferation through the activation of extracellular signal-regulated kinase (ERK) and Akt as well as the modulation of the level of cell cycle-associated proteins [19,20]. Other factors, such as adenosine and vascular endothelial growth factor (VEGF), also increase the proliferation of DPC or hair growth by activation of ERK [21,22], whereas hepatocyte growth factor (HGF) induces the proliferation of melanoma cells by activation of p38 [23].

Unsaturated fatty acid-derived prostaglandins, including latanoprost, isopropyl unoprostone, and bimatoprost, which have similar structures to omega-3, -6 and -9 fatty acids, have been shown to increase hair growth in mice or humans [24,25]. Studies on the reduction of hair loss using natural materials such as plants and seaweed containing unsaturated fatty acids have been conducted, with some studies showing that fermented foods such as cheese and yogurt are involved in regulation of gastric ulcer and inflammation [26,27]. Mackerel-derived fermented fish oil (FFO) contains omega-3 fatty acids, including docosahexaenoic acid (DHA) and eicosapentaenoic acid (EPA) [28]. FFO is composed of about 57% unsaturated fatty acids and 40.8% saturated fatty acids, and DHA (7.4%) and EPA (5.4%) of FFO were reduced compared to those of unfermented fish oil. Nevertheless, DHA is the second most abundant unsaturated fatty acid in FFO [29].

While various effects of FFO containing DHA have been revealed in some studies for ameliorations of atopic dermatitis, memory loss and oxidative stress [29,30,31], reduction of hair loss using fermented materials have been rarely reported. We therefore investigated the effects and action mechanisms of FFO and DHA on hair growth.

## 2. Results

### 2.1. FFO Extract Increased the Hair-Fiber Length of Rat Vibrissa Follicles

**Figure 1:**
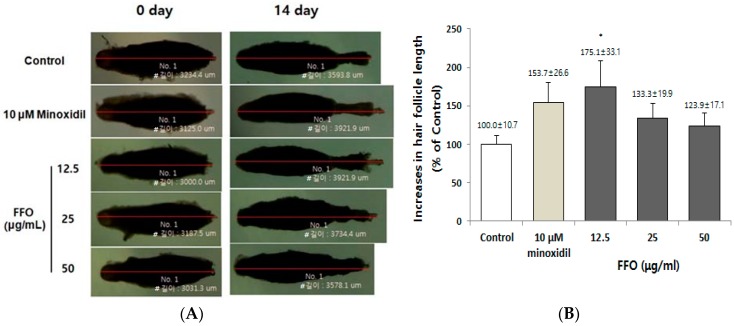
FFO extract increased the length of vibrissa follicles ex vivo. The rat vibrissa follicles were cultured with the indicated concentrations of FFO extract or minoxidil, a positive control, for 14 days. (**A**) Photographs of the length of the vibrissa follicles being cultured on Days 0 and 14. (**B**) Change in length of vibrissa follicles cultured in the presence of FFO extract or minoxidil for 14 days after isolation. The bar chart shows the percentage compared to the mean length of vehicle-treated control follicles at 14 days. Data are presented as mean ± SE. * *p* < 0.05 compared with the control. ^#^ means length.

To assess the hair-fiber elongation effect of FFO extract for hair growth, we first cultured rat vibrissa follicles, as described previously [32]. The rat vibrissa follicles were cultured in medium supplemented with FFO extract (12.5, 25, and 50 µg/mL) or minoxidil (10 µM) and were maintained for 14 days. On the 14th day after culture, FFO extract markedly increased the length of hair-fiber by 175.1% compared with the vehicle-treated control (100%) at a concentration of 12.5 µg/mL (Figure 1A,B). Minoxidil, a positive control, also showed an increase in the length of hair-fiber (153.1%), but to a lesser degree than that of FFO extract.

### 2.2. FFO Extract Stimulated the Telogen-to-Anagen Transition of the Hair-Cycle in Mice

**Figure 2:**
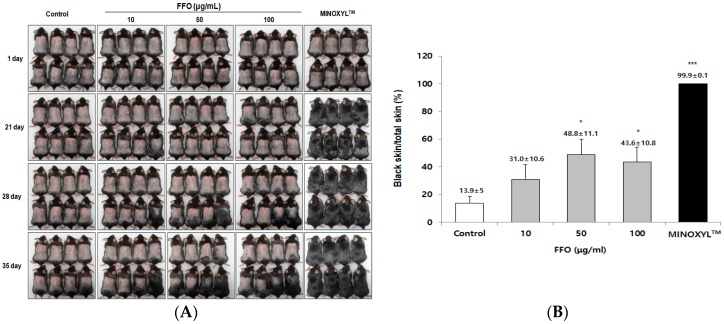
FFO extract accelerated hair cycle progression to anagen phase in C57BL/6 mice. FFO extract or MINOXYL^TM^ were topically applied to the backs of mice once a day for 35 days. (**A**) Photographs of the back skin taken every seven days. (**B**) The quantitative change of hair cycle in FFO-treated mice or MINOXYL^TM^-treated mice versus control mice, as calculated by dotmatrix planimetry. Data are presented as mean± SE (n = 8). * *p* < 0.05, *** *p* < 0.001 compared with the control.

To confirm the effect of FFO extract on hair growth in animal models, we used the established C57BL/6 mouse model, which is mainly used for in vivo hair growth studies [33,34]. C57BL/6 mice are known to have a change in skin color through the hair cycle, allowing for visual evaluation of the hair growth effect [35]. Briefly, as the hair cycle progresses from telogen to anagen, the skin color of C57BL/6 mice changes from pink to black. When FFO extract was topically applied to mice daily for 35 days after depilation, FFO extract was found to significantly stimulate hair growth (Figure 2A,B). Mice treated with FFO extracts (50 and 100 μg/mL) showed changes in skin color and hair growth from Day 28, and from Day 35, hair growth was observed to be significantly greater in the FFO-treated groups (48.8% and 43.6%) than in the control (13.9%) (Figure 2A,B). As previously reported [20], MINOXIL^TM^ (5% minoxidil) induces hair growth early, and from Day 21, hair growth was observed in all mice in the MINOXIL^TM^-treated group (Figure 2A,B). These results indicate that FFO extract stimulated the transition to anagen, as well as increased the hair-fiber length of the vibrissa follicles.

### 2.3. FFO Extract Increased the Proliferation of DPC through the Progression of Cell Cycle

**Figure 3:**
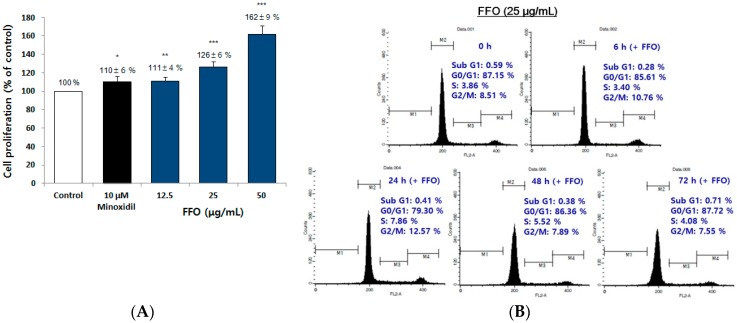
FFO extract increased the proliferation of dermal papilla cells (DPC). (**A**) Cell proliferations of DPC exposed to FFO extract (12.5, 25 and 50 μg/mL) are shown at 72 h. Minoxidil was used as a positive control. Data are presented as the mean ± standard deviation (SD). * *p* < 0.05, ** *p* < 0.01, *** *p* < 0.001 vs. vehicle-treated control. (**B**) The time course change of cell cycle by FFO extract in DPC at the indicated time.

It is known that dermal papilla plays an important role in the regulation of hair cycle and that cell proliferation is increased by minoxidil, a hair-growing drug [15,19]. To investigate whether the in vivo and ex vivo hair growth effects of FFO extract were caused by the proliferation of dermal papilla in hair follicles, we investigated the proliferation effect of immortalized DPC by FFO extract. The proliferation of cultured DPC for 72 h in media containing FFO extract (12.5, 25 and 50 μg/mL) was significantly increased compared to vehicle-treated controls (Figure 3A). In particular, the proliferation effect of DPC by FFO extract (25 and 50 μg/mL) was higher than that of the minoxidil used as a positive control (Figure 3A). These results suggest that FFO extract stimulates hair growth by controlling proliferation of DPC, such as minoxidil. Several studies have shown that cell proliferation and cell death are accompanied by cell cycle progression or arrest [36,37]. We thus investigated whether FFO extract induced the progression of cell cycle. When DPC were treated with 25 μg/mL FFO extract for 0, 6, 24, 48 and 72 h, the number of cells in S-phase (7.86%) and G2/M-phase (12.57%) increased compared to the control (3.86% and 8.51%, respectively), whereas the number of cells in G1-phase (79.3%) was decreased compared to the control (87.15%) (Figure 3B). These results indicate that FFO extract induced the proliferation of DPC via promoting cell cycle progression. These results suggest that FFO extract showed hair-growing effects by increasing the proliferation of DPCs through the progression of cell cycle.

### 2.4. FFO Extract Activated the Akt and MAPK Signaling in DPC

**Figure 4:**
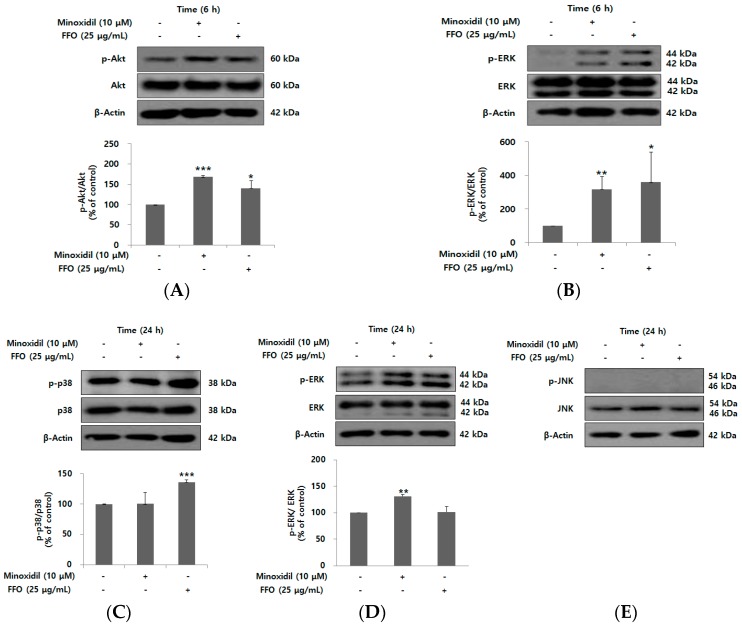
The effect of FFO extract on the level of MAPK and PI3K/Akt signaling proteins. (**A**–**E**) The DPC were stimulated with minoxidil (10 μM) or FFO extract (25 μg/mL) for 6 h or 24 h. Immunoblotting for vehicle, FFO extract and minoxidil-treated DPC lysate shows differential expression of phospho-Akt, phospho-ERK, phospho-p38 and phospho-JNK. β-Actin was used to confirm the same loading of proteins. Graphs represent the quantitative level of the proteins. The data are presented as the mean ± SD from three independent experiments. * *p* < 0.05, ** *p* < 0.01, *** *p* < 0.001 vs. vehicle-treated control.

Proliferation of DPC by minoxidil has also been shown to be due to Akt activation with PI3K/Akt being associated with an anti-apoptotic effect [19,38]. To examine whether the proliferative effect of FFO extract was induced by activation of PI3K/Akt signaling proteins, DPC were stimulated with 25 μg/mL FFO extract for 6 h. The level of phospho-Akt was significantly increased by FFO extract (Figure 4A). The mitogen-activated protein kinase (MAPK) signaling pathway, including ERK, p38 and c-Jun N-terminal kinase (JNK), regulates diverse cellular responses, such as cell proliferation and death [39]. We thus examined the changes in the phosphorylation of ERK, p38 and JNK after FFO extract treatment for 6 or 24 h. As shown in Figure 4B–E, FFO extract significantly increased the level of phospho-ERK at 6 h and phospho-p38 at 24 h, but did not affect the level of phospho-JNK in DPC. Minoxidil showed similar results to those reported by Kim et al., where the proliferation of human DPC was increased through the activation of Akt and ERK [19].

### 2.5. FFO Extract Activated Wnt/β-catenin Signaling in DPC

**Figure 5:**
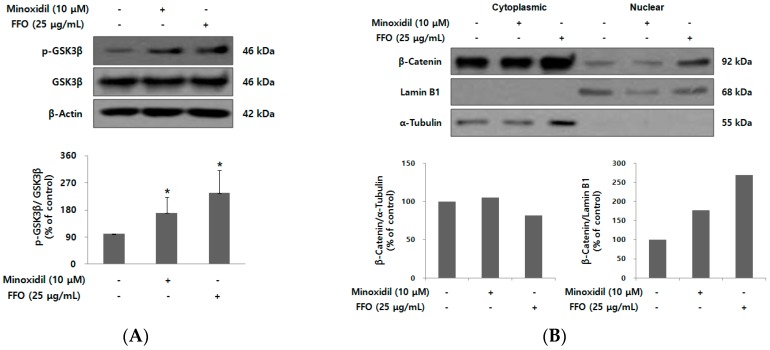
The effect of FFO extract on the level of Wnt/β-catenin signaling proteins. (**A**) DPCs were stimulated with minoxidil (10 μM) or FFO extract (25 μg/mL) for 18 h. The levels of phospho-GSK3β and GSK3β were determined using an immunoblot. The graph represents the quantitative level of the proteins. The data are presented as the mean ± SD from three independent experiments. * *p* < 0.05 vs. vehicle-treated control. (**B**) DPCs were stimulated with minoxidil (10 μM) or FFO extract (25 μg/mL) for 56 h. Cytoplasmic and nuclear fractions were prepared as described in Section 4. The protein level of β-catenin was determined using an immunoblot. α-Tubulin and lamin B1 were used to confirm the same loading of cytoplasmic and nuclear proteins. The graph represents the quantitative level of the proteins.

Hair growth is regulated by various factors, with activation of wnt/β-catenin signaling playing an important role in the regeneration of hair follicles and maintenance of anagen in the hair cycle [15,40]. Activation of wnt/β-catenin signaling induces the stabilization of β-catenin by phosphorylation/inactivation of GSK3β, which then regulates the expression of the target gene by promoting nuclear translocation of β-catenin [41]. To investigate whether FFO extract increased the proliferation of DPC by activating wnt/β-catenin signaling, an immunoblot assay was used. As shown in Figure 5A, FFO extract markedly increased the level of phospho-GSK3β. Next, we examined whether FFO extract could activate the nuclear translocation of β-catenin following induction of increased phosphorylation of GSK3β. After treatment of DPC with FFO extract for 56 h, β-catenin level was examined in both cytoplasmic and nuclear proteins. FFO extract was observed to increase nuclear β-catenin level in DPC (Figure 5B), indicating that FFO extract may increase the proliferation of DPC by the nuclear translocation of β-catenin through inactivation of GSK3β.

### 2.6. DHA Increased the Proliferation of DPC

**Figure 6:**
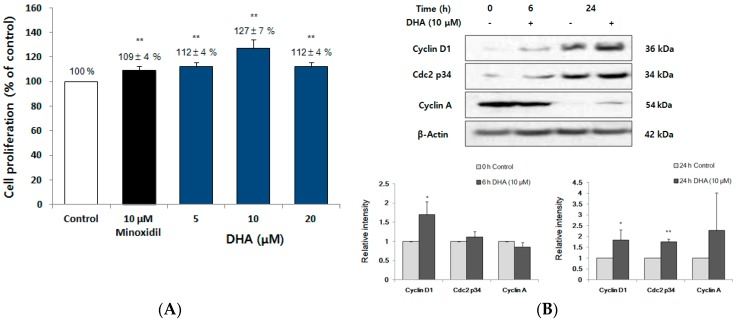
The effect of DHA on the proliferation of DPC. (**A**) Cell proliferation of DPC exposed to DHA (5, 10 and 20 μM, 72 h) is shown at 72 h. Minoxidil was used as a positive control. Data are presented as the mean ± SD. ** *p* < 0.01 vs. vehicle-treated control. (**B**) Immunoblot analysis of cell cycle-associated proteins on DPC stimulated in the presence or absence of 10 μM DHA. The cell lysate was analyzed by immunoblot using anti-cyclin D1, cyclin A and Cdc2 p34 antibodies. The graph represents the quantitative level of the proteins. * *p* < 0.05, ** *p* < 0.01 vs. vehicle-treated control.

To investigate whether DHA, a main component of the omega-3 fatty acids of FFO extract, induced the proliferation of the DPC, we examined the effect of DHA on the proliferation of DPC by MTT assay. The DPCs were stimulated with vehicle, DHA (5, 10, and 20 μM) or minoxidil (10 μM) for 72 h. DHA significantly increased the proliferation of DPC by 112%, 127% and 112% at the concentration of 5, 10 and 20 μM, respectively (Figure 6A). The increase of cell proliferation is accompanied by changes in dynamic cell cycle-associated proteins, including cyclins and CDKs [18,36]. To investigate whether DHA regulated the level of cell cycle-associated proteins, in the same way as it does for the DPC proliferation (Figure 6B), DPC were stimulated with 10 μM DHA for 0, 6 and 24 h, and the levels of cell cycle-associated proteins including cyclin D1, cdc2 p34 and cyclin A were examined. At 24 h, DHA was seen to increase levels of cyclin D1, cdc2 p34 and cyclin A (Figure 6B).

## 3. Discussion

Recently, quality of life has been emphasized more than in the past. While there is no significant impact on health, demand for new treatments for hair loss is growing due to increased interest in appearance. Although FFO is known to exhibit several biological activities such as anti-atopic dermatitis and anti-Alzheimer’s disease as a source of various fatty acids [28,29,30], its effect in the promoting of hair growth has not been investigated. In this study, we observed that FFO extract not only increased ex vivo cultured hair-fiber length, but also accelerated the transition to anagen phase in vivo. In addition, we demonstrated that FFO extract increased the proliferation of DPC by activating of wnt/β-catenin, ERK, p38, Akt and promoting cell cycle progression. Moreover, we discovered that DHA, a main omega-3 fatty acid of FFO extract, had a proliferative effect and cell-cycle-progression effect via the increases of cyclin D1 and cdc2 p34 on DPC.

The ex vivo culture of follicles does not fully reflect in vivo environments, but it is a miniorgan commonly used as an experimental model to test the effects on hair growth as it is composed of several cell types, including dermal papilla cells, matrix cells and stem cells [42]. It is used as a model as it is easy to see which cells proliferate or die, and changes in the expression of specific proteins [43]. In this study, we observed that vibrissa follicles cultured with FFO extract (12.5 µg/mL) could promote hair growth, supporting the effect of minoxidil reported in previous studies [20]. However, the concentrations of FFO extract that were significantly effective ex vivo vibrissa follicle culture were lower than those that were most effective in vivo and in vitro results. This concentration difference is thought to be due to the FFO extract added to the culture media changed every three days during follicle culture or as a result of interactions between various cells within the follicles, as described in our previous reports [20,44]. The skin color of C57BL/6 mice changes from pink (telogen) to black (anagen), leading to frequent use of this model in hair cycle and hair growth studies [34]. As expected, it was found that hair growth in areas of mouse skin topically applied with FFO extract was significantly greater than that of the control mice (Figure 2A,B). These ex vivo and in vivo results suggest the potential of FFO as a new material for inducing hair growth and treating hair loss.

DPC are always present, irrespective of changes in the hair cycle between anagen, catagen, and telogen [14,45]. Since the interaction between DPC and other cells in the hair follicle is an essential process for hair growth and hair follicle development, the control of DPC proliferation is important in evaluating hair-growth treatments [15,44,45]. Cell proliferation is also determined by cell cycle transition regulation at specific points such as G1, S and G2/M phases [18]. One of the important proteins involved in cell proliferation is cyclin D1, which is involved in the initiation of DNA synthesis. Several studies on cell cycle regulation have reported that cyclin A accumulates in the S phase and cdc2 p34 is required for progression to the G2/M phase, leading to cell cycle progression and cell proliferation [36,46]. In this study, FFO and DHA increased the proliferation of DPC (Figure 3A and Figure 5A). Our results showed that the number of cells of S phase and G2/M phase of the cell cycle were increased with the increase of DPC proliferation by FFO extract (Figure 3). The levels of cyclin D1 and cdc2 p34 were increased by DHA (Figure 6B) and these results are similar to flow cytometry results for FFO extract (G1 decrease, S and G2/M phase increase) (Figure 3B). These results suggest that FFO and DHA seem to increase cell proliferation through regulating cell cycle progression and cell cycle protein levels.

Akt, a serine/threonine protein kinase, is known to be a major regulator of cell proliferation in many types of cells by inhibition of apoptosis [47]. Akt kinase has been shown to initiate cell cycle progression by the inactivation of GSK3β, a mediator of Wnt/β-catenin signaling, which inhibits the degradation of cyclin D1 [48]. As shown in Figure 4A, the level of phospho-Akt was significantly increased by the FFO extract as expected from the increase of cyclin D1 level by DHA (Figure 6B) as well as promoting cell cycle progression by FFO extract (Figure 3B). Our findings are consistent with previous reports that indicate GSK3β is inactivated through phosphorylation of Akt and thereby induces the nuclear translocation by stabilization of β-catenin [41,49]. The MAPK family, consisting of ERK, p38, and JNK, are also kinases that play an important role in the regulation of cell proliferation [39]. Activation of p38 has a negative effect such as mitotic arrest and cyclin D1 reduction in cell proliferation [39], while p38 inhibitors inhibited melanoma cell proliferation by HGF in another report [23]. Interestingly, our results show that phospho-p38 level is increased by FFO extract, suggesting that activation of p38 by FFO may increase the proliferation of DPC.

Wnt/β-catenin plays an important role in various aspects of hair follicle development, such as regeneration and maintenance of anagen phase, and is considered to be an important signaling pathway for proliferation and differentiation in hair follicle cells [15,17,40,50]. Mice lacking β-catenin in keratinocyte expressing keratin 14 inhibited hair cycle progression and keratinocyte differentiation in hair follicles [17]. Prominin-1/CD133 is known to be one of the specific markers of DPC, and so prominin-1/CD133(+)DPC are mixed with epithelial cells to induce the production of new hair when transplanted into nude mice [51]. Overexpression of stabilized β-catenin in prominin-1/CD133-positive DPC not only increases the proliferation and differentiation of matrix keratinocyte but also increases the proliferation of DPC [52]. FFO extract increased nuclear translocation of β-catenin through the inactivation of GSK3β, as expected (Figure 5). These results suggest that FFO extract can induce hair growth through increasing the proliferation of DPC by activating the Wnt/β-catenin signaling pathway. However, since only the effects of FFO associated with the minoxidil mechanism have been observed, further studies are needed to determine whether FFO inhibits 5α-reductase activity, like finasteride’s mechanism of action.

In conclusion, we found that the hair growth promoting effect of FFO was mediated by the increase of DPC proliferation. FFO extract has been shown to increase the proliferation of DPC, the regulators of hair growth, by promoting cell cycle progression, and activating the Akt, ERK, p38 and wnt/β-catenin signaling pathways. In addition, DHA, a main omega-3 fatty acid of FFO, has the potential to promote the proliferation of DPC by modulating the level of cell cycle-related proteins. Our results could help development of hair loss treatments using FFO containing omega-3 fatty acids such as DHA.

## 4. Materials and Methods

### 4.1. Reagents

Dimethyl sulfoxide (DMSO), Earle’s balanced salt solution (EBSS), hydrocortisone, insulin, minoxidil, phosphate-buffered saline (PBS), phenylmethylsulfonylfluoride (PMSF), 3-[4,5-Dimethylthiazol-2-yl]-2,5-diphenyltetrazolium bromide (MTT) and docosahexaenoic acid (DHA) were purchased from Sigma-Aldrich (St. Louis, MO, USA). l-glutamine, antibiotic solution (Pen Strep) and Williams medium E were purchased from Gibco (Gibco Life Technologies, Grand Island, NY, USA). Dulbecco’s modification of Eagle’s medium (DMEM) and Fetal bovine serum (FBS) were purchased from Hyclone (Logan, UT, USA). NE-PER nuclear and cytoplasmic extraction reagents were purchased from Pierce Biotechnology, Inc. (Rockford, IL, USA). West-zol^TM^ Plus reagents were purchased from iNtRON (Seoul, Korea). X-ray film was purchased from Agfa-Gevaert (Mortsel, Belgium). Five percent minoxidil (MINOXIL^TM^) was purchased from Hyundai Pharm. Co. Ltd (Cheonan, Korea).

### 4.2. Preparation of Fermented Fish Oil (FFO) Extract

FFO was provided by Choung Ryong Fisheries Co., LTD (Jeju, Korea). The FFO was prepared from crushed mackerel by-product under anaerobic fermentation conditions. In detail: crushed mackerel by-product was fermented with *Lactobacillus plantarum*, *Saccharomyces cerevisiae* and sugar under anaerobic condition for 14 days. To isolate the oil fraction, hexane was added to the fermented mackerel by-product, and then concentrated using a vacuum evaporator. The FFO extract was dissolved in a 1:1 ratio of DMSO/EtOH (D/E solution) at a concentration of 50 mg/mL, and the D/E solution was used at 0.2% or less of the volume of the culture medium.

### 4.3. Animals

All animals were cared for using protocols (Approval number: 20160044) approved by the Institutional Animal Care and Use Committee (IACUC) of Jeju National University (Approval Date: 29 September 2016). Female 6-week-old C57BL/6 mice and male 3-week-old Wistar rats were purchased from Orient Bio (Seongnam, Gyeonggi, Korea) and provided with a standard laboratory diet and water ad libitum.

### 4.4. Isolation and Culture of Rat Vibrissa Follicles

Mystacial pads were isolated from the face of Wistar rats sacrificed by carbon dioxide using mayo scissors and washed three times with E/P buffer (1:1 mixture of EBSS and PBS supplemented with 1% Pen Strep). Anagen follicles were selected under a microscope and then isolated from mystacial pads using a sterile blade and tweezer under a stereomicroscope (Olympus, Tokyo, Japan) [32]. During the isolation of vibrissa follicles, they were carefully handled so as not to dry or damage. The isolated vibrissa follicles were transferred to a 24-well plate in Williams medium E supplemented with 50 nM hydrocortisone, 10 µg/mL insulin, 2 mM l-glutamine and 1% Pen Step. The isolated vibrissa follicles were cultured with FFO extract (12.5, 25 and 50 µg/mL) or 10 µM minoxidil at 37 °C in a 5% CO_2_/95% air. The culture medium containing the FFO extract or minoxidil was changed every 3 days and cultured for 14 days. The follicle length was measured once every 7 days with DP controller software ver. 1.1.1.65 (Olympus, Tokyo, Japan), and the change of follicle length was compared to the vehicle-treated control.

### 4.5. Hair Growth Activity In Vivo

As previously described, the removal of hair from the back skin in C57BL/6 mice induced anagen phase of hair cycle [35]. The female C57BL/6 mice (P49) were anesthetized with ketamine-xylazine, and the back skin of mice was shaved using animal clipper. The mice were observed 2 days after hair removal and wounded mice were excluded. The FFO extract (10, 50 and 100 µg/mL) or MINOXIL^TM^, a positive control, were topically applied every day for 35 days. The mice were photographed once every 7 days for 35 days while observing changes in their skin color. The changes in hair growth were quantified as a percentage compared to the vehicle-treated control group using dotmatrix planimetry [53].

### 4.6. Cell Viability Assay

Rat vibrissa immortalized DPC were provided by the Skin Research Institute, Amore Pacific Corporation Research and Development Center, Korea. The DPC were cultured in DMEM supplemented with 10% FBS and 1% Pen Strep at 37 °C in a humidified atmosphere under 5% CO_2_. Cell viability of DPC was estimated by measuring the metabolic activity using a MTT assay. Briefly, DPC (5.0 × 10^4^ cells/mL) were seeded onto 24 well plate, cultured for 24 h under serum starvation containing 1% FBS, and then treated with 2 µL of vehicle (D/E solution) as a control, FFO extract (12.5, 25 and 50 µg/mL), DHA (5, 10 and 20 µM), or minoxidil (10 µM) for 72 h. After incubation, 250 µL of MTT (2 mg/mL) stock solution per well was added and incubated at 37 °C for 4 h. Next, the media was carefully aspirated and 500 µL of DMSO was added to each well to dissolve the formazan crystals. The dissolved solution was transferred at 200 µL per 96 well plate and the absorbance of the plate was immediately measured at 540 nm using a microplate reader (Bio Tek Instrument Inc., Winooski, VT, USA). All experiments were repeated three times, and the mean value of the measured absorbance was calculated. The results are expressed by comparing the percentage of absorbance changed by treatment with FFO extract, DHA, or minoxidil compared to vehicle-only control.

### 4.7. Cell Cycle Analysis

DPC (1.0 × 10^6^ cells/100-mm dish) were incubated for 24 h in 1% serum condition, and then treated with FFO extract (25 µg/mL) for 0, 6, 24, 48 and 72 h. The cells were fixed with 70% ethanol and washed with two times with PBS. The cells were stained with propidium iodide (10 μg/mL) in the presence of RNAse (50 μg/mL) for 30 min at 25 °C. The DNA content of the cells were analyzed by FACSCalibur equipped with Cell Quest Software (Becton-Dickinson, San Jose, CA, USA).

### 4.8. Western Blot Analysis

Western blot analysis was performed as previously described. DPC were seeded onto 100 mm plates at a density of 1.0 × 10^6^ cells per plate. Cells were pre-incubated with DMEM containing 1% FBS for 24 h and then treated with FFO extract (25 µg/mL) or minoxidil (10 µM) for 6, 18, 24, or 48 h. Cells were washed twice with ice-cold PBS and then the whole cell lysates were isolated using PRO-PREP protein extraction solution (iNtRON Biotechnology, Seoul, Korea). After centrifugation at 21,000× *g* for 15 min at 4 °C, the supernatant of cell lysate was obtained. The intracellular fractions (nuclear and cytoplasmic) were obtained using NE-PER kit according to the manufacturer’s protocol. All of the separated proteins were stored at −70 °C until the experiment, and proteins were quantified using the Bradford assay. The proteins (20 µg) of total lysates, nuclear fraction or cytoplasmic fraction were subjected to 10–12% SDS-polyacrylamide gel electrophoresis and transferred onto PVDF membranes. The membranes were blocked with 5% nonfat dry milk diluted in T-TBS (0.1% Tween-20, 50 mM Tris, pH 7.6, 150 mM NaCl) for 2 h, and incubated with specific primary antibodies (Table 1) at 4 °C overnight. After washing six times with T-TBS for 30 min (each wash for 5 min), blots were incubated with horseradish peroxidase labeled anti-mouse IgG or anti-rabbit IgG secondary antibodies at room temperature for 1 h. Blots were washed six times with T-TBS for 30 min before being exposed to West-zol^TM^ Plus reagents. An enhanced chemiluminescent detection system was used according to the manufacturer’s protocol, and immunoblots were exposed to X-ray films. Band intensities were quantified using NIH image software (http://rsb.info.nih.gov/ij).

### 4.9. Statistical Analysis

Data are expressed as the mean ± standard deviation (SD) or standard error (SE) of at least triplicate experiments. The statistical significance between the experimental and control groups was determined by the student’s *t*-test using SigmaStat Software ver. 3.5 (San Jose, CA, USA). *p* < 0.05 was considered to be statistically significant.

## Figures and Tables

**Table 1:** List of antibodies used for immunoblotting.

Antibodies	Supplier	Species	dilution
phospho(Ser473)-Akt	Cell Signaling	Rabbit	1:1000
Akt	Cell Signaling	Rabbit	1:1000
phosho(Thr202/Tyr204)-ERK1/2	Cell Signaling	Rabbit	1:1000
ERK1/2	Cell Signaling	Rabbit	1:1000
phospho(Thr183/Tyr185)-JNK	Cell Signaling	Mouse	1:1000
JNK	Cell Signaling	Rabbit	1:1000
phospho(Thr180/Tyr182)-p38	Cell Signaling	Rabbit	1:1000
p38	Cell Signaling	Rabbit	1:1000
β-catenin	Santa Cruz	Rabbit	1:1000
phospho(Ser9)-GSK3β	Cell Signaling	Rabbit	1:1000
GSK3β	Cell Signaling	Rabbit	1:1000
Lamin B1	Abcam	Rabbit	1:2000
α-Tubulin	Santa Cruz	Mouse	1:250
Cyclin D1	BD Biosciences	Mouse	1:1000
Cdc2 p34	Santa Cruz	Mouse	1:1000
Cyclin A	Santa Cruz	Rabbit	1:1000
β-actin	Sigma-Aldrich	Mouse	1:5000
